# Iron Metabolism in Ferroptosis

**DOI:** 10.3389/fcell.2020.590226

**Published:** 2020-10-07

**Authors:** Xin Chen, Chunhua Yu, Rui Kang, Daolin Tang

**Affiliations:** ^1^Guangzhou Municipal and Guangdong Provincial Key Laboratory of Protein Modification and Degradation, The Third Affiliated Hospital, School of Basic Medical Sciences, Guangzhou Medical University, Guangzhou, China; ^2^Department of Surgery, UT Southwestern Medical Center, Dallas, TX, United States

**Keywords:** ferroptosis, cell death, iron, lipid perioxidation, disease

## Abstract

Ferroptosis is a form of regulated cell death that is characterized by iron-dependent oxidative damage and subsequent plasma membrane ruptures and the release of damage-associated molecular patterns. Due to the role of iron in mediating the production of reactive oxygen species and enzyme activity in lipid peroxidation, ferroptosis is strictly controlled by regulators involved in many aspects of iron metabolism, such as iron uptake, storage, utilization, and efflux. Translational and transcriptional regulation of iron homeostasis provide an integrated network to determine the sensitivity of ferroptosis. Impaired ferroptosis is implicated in various iron-related pathological conditions or diseases, such as cancer, neurodegenerative diseases, and ischemia-reperfusion injury. Understanding the molecular mechanisms underlying the regulation of iron metabolism during ferroptosis may provide effective strategies for the treatment of ferroptosis-associated diseases. Indeed, iron chelators effectively prevent the occurrence of ferroptosis, which may provide new approaches for the treatment of iron-related disorders. In this review, we summarize recent advances in the theoretical modeling of iron-dependent ferroptosis, and highlight the therapeutic implications of iron chelators in diseases.

## Introduction

Despite its essential role in life, excessive iron is toxic due to its ability to generate reactive oxygen species (ROS) and to even trigger cell death. There are two main distinct categories of cell death, namely accidental cell death (ACD) and regulated cell death (RCD) (Galluzzi et al., 2018). ACD is usually due to irreparable damage after external or internal physical and chemical stimulation, and it occurs in an uncontrollable way, whereas RCD involves a precise signaling pathway controlled by designated cellular machinery. Compared with ACD, RCD has received extensive attention due to it being closely related to pathologic conditions and human diseases, which can be targeted by small molecule compounds or drugs. A growing number of RCD types have been proposed, such as necroptosis, pyroptosis, ferroptosis, and alkaliptosis. They may share similar necrotic morphology, but their molecular mechanisms are different (Tang et al., 2019).

Ferroptosis is a type of oxidative cell death that is induced by the accumulation of iron-mediated lipid peroxidation (Xie et al., 2016a; Stockwell et al., 2017). Its name was coined in 2012 as the discovery of small molecules (e.g., erastin and RSL3) selectively induced a non-apoptotic form of RCD in cancer cells that can be blocked by iron chelators or lipophilic antioxidants (e.g., vitamin E/α-tocopherol and ferrostatin-1) (Dixon et al., 2012). The morphological features of ferroptosis are distinct from apoptotic cell death. Apoptotic cells are characterized by membrane blebbing, cellular shrinkage, nuclear fragmentation, and chromatin condensation, whereas ferroptotic cells show typical necrotic morphology, such as an incomplete plasma membrane and the release of intracellular contents, especially damage-associated molecular patterns (DAMPs) (Wen et al., 2019). Ultrastructural analysis further revealed that the mitochondria of ferroptotic cells show a loss of structural integrity and smaller size (Dixon et al., 2012), whereas mitochondria are usually swollen in apoptotic cells (Tang et al., 2019).

Autophagy is a lysosome-dependent degradation system through which cells break down various cargos, such as organelles and proteins. There is a complex interaction between autophagy and lipid metabolism (Xie et al., 2020). Although an early study showed that ferroptosis is unrelated to autophagy, increased autophagy flux is indeed widely observed in the ferroptotic response (Zhou et al., 2019; Liu J. et al., 2020). While autophagy plays an adaptive role in protecting cells from environmental stress, it can promote ferroptosis in many cases (Zhou et al., 2019; Liu J. et al., 2020). Specifically, selective autophagy (e.g., ferritinophagy, lipophagy, and clockophagy) is involved in mediating ferroptotic cell death through the degradation of anti-ferroptosis regulators (Gao et al., 2016; Hou et al., 2016; Bai et al., 2019; Yang et al., 2019). Autophagy regulators, such as beclin 1 (BECN1) (Song et al., 2018), mechanistic target of rapamycin kinase (MTOR) (Liu Y. et al., 2020), and a lysosomal protease cathepsin B (CTSB) (Gao et al., 2018) contribute to ferroptotic cell death through affecting lipid peroxidation. Since there are many different types of selective autophagy, the relationship between autophagy and ferroptosis in regulating cell fate needs further investigation (Kang and Tang, 2017).

In this review, we first introduce the molecular mechanisms of ferroptosis and then focus on a discussion of regulators of iron metabolism in ferroptosis (Table 1). Finally, we describe the therapeutic implications of iron chelators in the damage and diseases associated with ferroptosis.

**TABLE 1:** Main proteins modulating iron metabolism in ferroptosis.

Gene	Description	Functions	Diseases/models	References
CISD1	CDGSH iron sulfur domain 1	Regulates mitochondrial iron homeostasis	Cancer	Yuan et al., 2016a
CISD2	CDGSH iron sulfur domain 2	Regulates mitochondrial iron homeostasis	Cancer	Kim et al., 2018
CP	Ceruloplasmin	Mediates oxidation of Fe^2+^ to Fe^3+^	Cancer, ischemic stroke	Shang et al., 2020; Tuo et al., 2017
FTH1	Ferritin heavy chain 1	Stores iron	*Drosophila*	Mumbauer et al., 2019
FTL	Ferritin light chain	Stores iron	*Drosophila*	Mumbauer et al., 2019
FTMT	Ferritin mitochondrial	Stores iron	Neuronal cells	Wang et al., 2016
HMOX1	Heme oxygenase 1	Catabolizes heme	Cancer, ferroptotic damage in heart and renal proximal tubular cells	Kwon et al., 2015; Chang et al., 2018; Fang et al., 2019; Adedoyin et al., 2018; Sun et al., 2016b
HSPB1	Heat shock protein family B small member 1	Inhibits iron uptake	Cancer	Sun et al., 2015
IREB2	Iron-responsive element binding protein 2	Regulates the translation of mRNAs that affect iron homeostasis	Cancer	Dixon et al., 2012
LTF	Lactotransferrin	Transports iron	Cancer	Wang et al., 2020
NCOA4	Nuclear receptor coactivator 4	Mediates ferritinophagy	Cancer, chronic obstructive pulmonary disease, aging, liver fibrosis	Hou et al., 2016; Gao et al., 2016; Yoshida et al., 2019; Masaldan et al., 2018; Kong et al., 2019; Chen G.Q. et al., 2020
NFS1	NFS1 cysteine desulfurase	Mediates biosynthesis of the Fe-S cluster	Cancer	Alvarez et al., 2017
PCBP1	Poly(RC) binding protein 1	Iron chaperones	Hepatic steatosis	Protchenko et al., 2020
PROM2	Prominin-2	Promotes ferritin export	Cancer	Brown et al., 2019
SLC25A28	Solute carrier family 25 member 28	Mediates mitochondrial iron import	Hepatic stellate cells	Zhang et al., 2020
SLC39A14	Solute carrier family 39 member 14	Mediates iron uptake	Liver fibrosis	Yu et al., 2020
SLC40A1	Solute carrier family 40 member 1	Mediates iron export	Cancer, testicular ischemia-reperfusion injury	Geng et al., 2018; Li L. et al., 2018
TF	Transferrin	Transports iron	Cancer	Gao et al., 2015
TFRC	Transferrin receptor	Mediates iron uptake	Cancer	Gao et al., 2015; Ooko et al., 2015; Song et al., 2016; Wu et al., 2019; Yang and Stockwell, 2008

## Core Molecular Mechanisms of Ferroptosis

**FIGURE 1:**
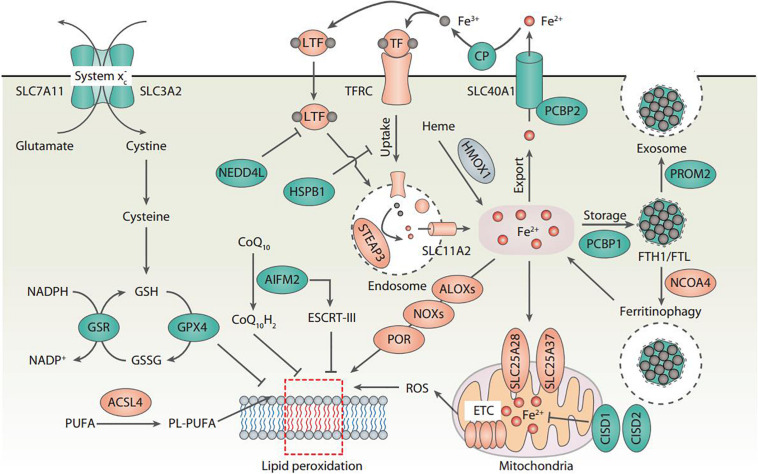
Core molecular mechanisms of ferroptosis. Ferroptosis is a type of oxidative cell death that can cause lipid peroxidation and membrane damage. Many oxidative and antioxidant pathways affect the ferroptotic reaction. In particular, system xc^–^-mediated cystine uptake and subsequent cysteine production are required for GSH biosynthesis, which further enhances the anti-lipid peroxidation activity of GPX4. The inhibition of SLC7A11 and GPX4 leads to the accumulation of iron-dependent lipid peroxidation, thus causing ferroptotic cell death. The activation of ALOX, NOX, or POR promotes lipid peroxidation, whereas the activation of the ESCRT-III complex repairs damaged membranes during ferroptosis. Importantly, all aspects of iron metabolism, including iron absorption, storage, export, and utilization, have an important regulatory effect on ferroptosis.

The central role of lipid peroxidation in driving ferroptotic cell death indicates that ferroptosis can be caused by the collapse of the glutathione (GSH)-glutathione peroxidase 4 (GPX4) antioxidant systems (Figure 1). System xc^–^ is a heterodimeric transmembrane complex composed of light chain, solute carrier family 7 member 11 (SLC7A11/xCT), and heavy chain, solute carrier family 3 member 2 (SLC3A2). After entering the cells by system xc^–^, cystine is quickly reduced to cysteine, which is mainly utilized for the synthesis of GSH. As a potent low molecular weight antioxidant in cells, GSH is utilized by GPX4, which uses highly nucleophilic selenocysteine to reduce lipid peroxides into lipid alcohols. The pharmacological inhibitors of system xc^–^ (e.g., erastin) and GPX4 (e.g., RSL3) are the classical two ferroptosis inducers. In addition, several GPX4-independent anti-ferroptosis pathways have recently been identified, such as the apoptosis-inducing factor mitochondria-associated 2 (AIFM2)-mediated CoQ10 production pathway (Bersuker et al., 2019; Doll et al., 2019), and the endosomal sorting complex required for transport-III (ESCRT-III)-dependent membrane repair pathway (Dai et al., 2020). These findings indicate that multiple antioxidants and membrane repair pathways limit the oxidative damage caused by ferroptosis, although their selectivity and specificity in ferroptosis are still unclear.

Lipid peroxidation results in the oxidation of polyunsaturated fatty acid (PUFA) of the membrane lipids. An impaired antioxidant system can cause or accelerate lethal lipid peroxidation, which is inhibited by various synthetic antioxidants (e.g., ferrostatin-1 and liproxstatin-1). Acyl-CoA synthetase long-chain family member 4 (ACSL4) is a crucial pro-ferroptotic regulator by catalyzing the synthesis of long-chain polyunsaturated CoAs, especially arachidonic acid, thus enriching cellular membranes with PUFA (Yuan et al., 2016b; Doll et al., 2017; Kagan et al., 2017). Several hypotheses have been proposed to explain the subsequent oxidation of PUFA after ACSL4-mediated PUFA synthesis (Figure 1). First, nicotinamide adenine dinucleotide phosphate (NADPH) oxidases (NOXs), a class of membrane-bound enzyme complexes that catalyzes the production of superoxides, contributes to iron-dependent accumulation of lipid peroxidation during ferroptosis (Dixon et al., 2012; Xie et al., 2017; Yang et al., 2020). Second, lipoxygenases (ALOXs) drive ferroptosis through directly catalyzing the oxygenation of PUFA (Yang et al., 2016; Kagan et al., 2017). Third, cytochrome P450 oxidoreductase (POR) coupled to cytochrome P450 (CYP) monooxygenases represents an alternative source of ROS for the induction of ferroptosis-related lipid peroxidation (Zou et al., 2020). Fourth, ROS generated by the electron leakage of the mitochondrial electron transport chain (ETC) may be another factor leading to lipid peroxidation in the process of ferroptosis in some cases (Gao et al., 2019). However, since NOXs, ALOXs, and CYP are all large family enzymes, it is difficult to determine which member of these enzymes is responsible for the ferroptosis.

## Regulators of Iron Metabolism in Ferroptosis

Although the exact mechanisms related to iron in ferroptosis remain obscure, there is no doubt about the key role of iron metabolism in the process of ferroptosis, because (1) Iron chelators block ferroptotic cell death *in vitro* and *in vivo* (Dixon et al., 2012); (2) Increased cellular labile iron is usually observed during the induction of ferroptosis (Hou et al., 2016); (3) An exogenous supplement of iron increases the sensitivity of cells to ferroptosis inducers (e.g., erastin) (Dixon et al., 2012); (4) Excessive heme and non-heme iron can directly induce ferroptosis (Li et al., 2017); (5) Several heme and non-heme iron-containing enzymes, such as ALOXs, NOXs, and CYP, are responsible for the production of lipid peroxidation (Dixon et al., 2012; Yang et al., 2016; Kagan et al., 2017; Xie et al., 2017; Yang et al., 2020; Zou et al., 2020); and (6) Iron-mediated ROS production by Fenton reaction promotes lipid peroxidation in ferroptosis (Dixon et al., 2012). Thus, multiple regulators of iron metabolism involved in iron uptake, storage, utilization, and efflux may impact the sensitivity of ferroptosis (Figure 1), which are discussed below.

### Transferrin and Lactotransferrin

Transferrin (TF) is a 76-kDa glycoprotein that tightly, but reversibly, binds to iron in the blood and transports it to various tissues and organs. TF contains two globular lobes at the N- and C-termini, each of which can bind to one ferric ion (Fe^3+^), as well as other metal ions (e.g., copper, zinc, and manganese) with different affinities. The iron-binding properties of TF are highly dependent on changes in pH. Iron efficiently binds to TF outside the cells at pH ∼7.4 and iron releases from TF when delivery to acidic endosomes (pH ∼5.5) by receptor-mediated endocytosis. Low serum levels of TF usually occur in patients with chronic inflammatory states or in hospitalized patients with poor clinical outcomes. When there is adequate nutrition, TF levels can be retained at a high level. In humans, due to homozygous or compound heterozygous mutations in TF genes, atransferrinemia is a rare autosomal recessive metabolic disorder. This disease is associated with microcytic anemia and hemosiderosis in the heart and liver. Hypotransferrinemic (Trf^hpx/hpx^) mice carry a spontaneous mutation linked to the TF locus (Bernstein, 1987). They are born alive, but die before weaning due to refractory iron-deficient hypochromic anemia. The Trf^hpx/hpx^ mice also survive when treated with serum or TF injections (Trenor et al., 2000).

Transferrin is required for the induction of ferroptotic cell death based on nutrient depletion experiments (Gao et al., 2015). For example, in the presence of serum, cell death induced by the deprivation of amino acids takes the form of ferroptosis, instead of apoptosis or necroptosis (Gao et al., 2015). This amino acid starvation-induced ferroptosis is probably due to cystine starvation and subsequent cellular GSH depletion. TF is essential for this amino acid starvation-induced ferroptosis (Gao et al., 2015). Serum without macromolecules (e.g., TF) fails to mimic the ferroptosis-inducing activity, while the addition of recombinant iron-saturated holo-TF induces cell death in the same condition (Gao et al., 2015). Consistently, iron-free apotransferrin (apo-TF) fails to produce cell death-inducing activity (Gao et al., 2015). Similarly, co-treatment with holo-TF significantly increases artesunate-induced ferroptosis in pancreatic cancer cells (Eling et al., 2015). These finding suggest that TF is a key positive regulator of ferroptotic cell death. However, hepatocyte-specific TF knockout mice are more susceptible to high-iron diet-induced ferroptotic liver fibrosis (Yu et al., 2020), indicating that hepatic TF plays a protective role in ferroptosis-induced liver fibrosis. Patients with liver cirrhosis have reduced levels of hepatic TF and increased levels of hepatic iron (Yu et al., 2020), supporting the idea of a protective role for hepatic TF in maintaining liver function.

Lactotransferrin (LTF) is a member of the TF family that is responsible for increased intracellular iron during chronic inflammation and tissue injury. Similar to TF, LTF functions as a promoter of ferroptosis in cancer cells (Wang et al., 2020). In contrast, LTF protein degradation that is mediated by neural precursor cell-expressed developmentally downregulated gene 4 (NEDD4)-like E3 ubiquitin protein ligase (NEDD4L) blocks iron-dependent lipid peroxidation during ferroptotic cancer cell death (Wang et al., 2020). These findings indicate that targeting ubiquitin-proteasome system-dependent LTF protein degradation may enhance the anticancer activity of ferroptosis-based therapy. The direct role of LTF-mediated ferroptosis in non-cancer cells and tissue injury remains to be further studied.

### Transferrin Receptor

Transferrin receptor (TFRC/TFR1) is a dimeric glycoprotein receptor for iron-loaded TF at the surface of plasma. The extracellular domains of TFRC have high affinity to bind diferric TF, as compared to monoferric TF or apo-TF. Following binding to TFRC, the TF-TFRC complex is internalized via receptor-mediated endocytosis. In the endosome (an acidic environment), the released iron needs to be reduced from Fe^3+^ to Fe^2+^ by transmembrane ferrireductase STEAP3. Once the iron is released into cytosol through solute carrier family 11 member 2 (SLC11A2/DMT1), TFRC and TF are recycled back to the cell surface and extracellular fluid, respectively. Transferrin receptor 2 (TFR2) is a homolog to TFRC, but is unrelated to iron transport and instead is linked to iron sensing. TFRC knockout mice exhibited early embryonic lethality, which is the result of impaired erythroid and neuronal development (Levy et al., 1999). A homozygous p.Tyr20His mutation in TFRC in human patients causes immunodeficiency combined with impaired development or function of T and B lymphocytes (Jabara et al., 2016). Both the patients and the TFRC^Y20H/Y20H^ mice had only mild anemia, which may be due to an accessory TfR1 endocytosis signal provided by STEAP3 (Jabara et al., 2016). Interestingly, the selective inactivation of TFRC in murine intestinal epithelial cells induces a severe disruption of the epithelial barrier, which is fully rescued by the enforced expression of a mutant allele of TFRC that is unable to serve as a receptor for TF (Chen et al., 2015). These findings suggest that, in some contexts, TFRC also plays a role independent of the classical function of iron absorption.

The increased expression of TFRC in malignant cells is mainly to meet the high requirement of iron for cell proliferation. Therefore, inducing ferroptosis in TFRC-expressed cancers is a potential cancer treatment strategy. In addition to cancer, reduced TFRC palmitoylation has been demonstrated to contribute to neurodegeneration accompanied by brain iron accumulation, while the antimalarial agent artesunate can reverse the abnormal TFRC palmitoylation in cultured fibroblasts of neurodegeneration subjects (Drecourt et al., 2018). Since artesunate has been shown to induce ferroptosis in cancer cells, whether TFRC palmitoylation also contributes to its antitumor activity of ferroptosis needs further validation (Eling et al., 2015). The expression of TFRC in cancer cells is positively correlated with the ferroptotic response induced by artemisinin derivatives (Ooko et al., 2015) or erastin (Song et al., 2016; Wu et al., 2019). For example, oncogenic RAS renders cells sensitive to erastin-induced ferroptosis through upregulating the expression of TFRC, thus enriching the cellular iron pool (Yang and Stockwell, 2008). The knockdown of TFRC also ameliorates erastin-induced ferroptosis in RAS mutation cells (Yang and Stockwell, 2008). Moreover, the knockdown of TFRC inhibits cystine starvation-triggered ferroptotic cell death (Gao et al., 2015), supporting the requirement of iron import for ferroptosis. The antibody of TFRC (3F3-FMA) was recently identified by screening monoclonal antibodies generated from immunizing mice with membrane fractions from piperazine erastin-treated cells (Feng et al., 2020). The accumulation of TFRC (3F3-FMA) is specific to ferroptosis, but not to apoptosis (Feng et al., 2020). Thus, in addition to ACSL4, TFRC may serve as a biomarker for the sensitivity of ferroptosis.

### Solute Carrier Family 39 Member 8, and Member 14

The SLC39/ZIP family is made up of transmembrane proteins that act as broad-scope metal ion transporters, mediating the uptake of a variety of nutritionally important divalent metals, such as zinc, iron, and manganese. Among the SLC39 family members, solute carrier family 39 member 14 (SLC39A14/ZIP14) and solute carrier family 39 member 8 (SLC39A8/ZIP8) are the most closely related transporters. They mediate the iron uptake through directly transporting non-transferrin-bound iron (NTBI) across the cell membrane. The knockout of SLC39A8 in mice causes a combination of stunted growth, severe anemia, and a dysregulation of hematopoiesis and organ development *in utero*, as well as neonatal lethality (Galvez-Peralta et al., 2012). SLC39A8^–/–^ newborns exhibit a decreased level of zinc and iron in several tissues (Galvez-Peralta et al., 2012). Mice lacking SLC39A14 show growth retardation and impaired gluconeogenesis, which may be due to impaired G-protein–coupled receptor (GPCR) signaling required for systemic growth. The loss of SLC39A14 in mice also markedly reduces the liver’s absorption of NTBI and prevents hepatic iron overload in mouse models of hemochromatosis (Jenkitkasemwong et al., 2015). As expected, the conditional knockout of hepatic SLC39A14 reduces iron accumulation in liver and ferroptosis-mediated liver fibrosis, indicating that SLC39A14-mediated iron uptake promotes ferroptotic liver injury and disease (Yu et al., 2020). Whether SLC39A8 plays a role similar to that of SLC39A14 in promoting ferroptosis *in vivo* remains to be further studied.

### Ferritin Heavy Chain 1 and Ferritin Light Chain

Ferritin is a cytosolic iron storage protein composed of two subunits, namely ferritin heavy chain 1 (FTH1) and ferritin light chain (FTL). Twenty-four ferritin subunits are assembled into a high molecular weight apoferritin shell, which can chelate up to approximately 4500 iron atoms. FTH1 has ferroxidase activity and can convert Fe^2+^ to Fe^3+^, which is important for subsequent iron entry into the ferritin mineral core, an event that is helped by FTL. The inactivation of FTH1 by homologous recombination in mice is embryonically lethal, whereas the knockout of FTL leads to embryonic lethality in approximately 50% of newborn mice (Li et al., 2015). These findings indicate a different role of FTL and FTH1 in embryonic development. FTL knockout mice exhibit systemic and brain iron dyshomeostasis but no obvious signs of neurodegeneration (Li et al., 2015). Mutations in FTL in humans results in a neurodegenerative disease (namely hereditary ferritinopathy), which is characterized by ferritin-containing intracellular inclusion bodies and increased iron in the brain and other organ systems. Patients who lack FTL (but not FTH1) experience idiopathic generalized seizures and atypical restless leg syndrome, which may be due to increased ROS production and cell damage in fibroblasts (Cozzi et al., 2013).

In addition to cytosolic ferritin, the overexpression of mitochondrial ferritin (FTMT) in neuronal cells inhibits erastin-induced ferroptosis and increases the cellular labile iron pool (Wang et al., 2016). Consistently, wild-type flies (rather than FTMT overexpressing transgenic flies) died within 3 weeks after being fed an erastin-containing diet (Wang et al., 2016). The depletion of FTH1 in Drosophila larval wing disks leads to ferroptosis-associated severe growth defects, while the depletion of FTL causes only minor defects (Mumbauer et al., 2019). Although these findings indicate that ferritin has protective effects on ferroptosis in various models, the benefits of FTH1 and FTL on ferroptosis are not the same.

### Nuclear Receptor Coactivator 4

The degradation of ferritin can be completed by ferritinophagy, which is a type of selective autophagy mediated by nuclear receptor coactivator 4 (NCOA4). Like the knockdown of autophagy-related 5 (ATG5) or ATG7, the knockdown of NCOA4 also blocks ferritin degradation and suppresses erastin-induced ferroptosis in fibroblasts and pancreatic cancer cells, whereas the overexpression of NCOA4 promotes ferroptosis by degrading ferritin (Hou et al., 2016). These findings provide the first direct link between autophagy and ferroptosis. Increased ferritinophagy is also required for cystine starvation-induced ferroptotic cell death (Gao et al., 2016). Accordingly, the knockdown of ATG3, ATG13, BECN1, and microtubule-associated protein 1 light chain 3 beta (MAP1LC3B) limits cystine starvation- or erastin-induced ferroptosis by blocking ferritin degradation and iron accumulation in fibroblasts (Gao et al., 2016). In addition to classical ferroptosis activators, non-classical ferroptosis activators can contribute to ferroptotic cell death via ferritinophagy. Such activators include dihydroartemisinin, JQ1, siramesine/lapatinib, or salinomycin in various cancer cells, indicating a wider role of ferritinophagy in promoting ferroptotic cancer cell death (Zhou et al., 2019; Liu J. et al., 2020).

NCOA4-mediated ferritinophagy also plays a role in cigarette smoke-induced ferroptosis in lung epithelial cells (Yoshida et al., 2019), supporting a role of ferroptosis in the pathogenesis of cigarette smoke-induced chronic obstructive pulmonary disease. Ferritinophagy-mediated iron accumulation may regulate the process of aging in cells (Masaldan et al., 2018) and carbon tetrachloride (CCl4)-induced liver fibrosis in mice (Kong et al., 2019). These results further strengthen a link between ferritinophagy and ferroptosis in various diseases. Autophagy-independent lysosomal degradation of ferritin also promotes dihydroartemisinin-induced ferroptotic cancer cell death (Chen G.Q. et al., 2020), indicating an alternative mechanism leading to ferritin degradation during ferroptosis.

### Prominin-2

Prominin-2 (PROM2) is a member of the prominin family of pentaspan membrane glycoproteins, and is selectively associated with plasma membrane protrusions. Ferritin can be released into the extracellular space by PROM2-mediated exosomes, which drives ferroptosis resistance (Brown et al., 2019). RSL3-induced PROM2 upregulation is observed in ferroptosis-resistant cells (e.g., MCF10A and Hs578t), but not in ferroptosis-sensitive cells (e.g., MDA-MB-231) (Brown et al., 2019), indicating that the induction of PROM2 expression may be correlated with resistance to ferroptosis. The knockdown of PROM2 expression increases sensitivity to ferroptosis in ferroptosis-resistant cells, while the overexpression of PROM2 inhibits ferroptosis in ferroptosis-sensitive cells (Brown et al., 2019). Mechanistically, PROM2 localizes to multivesicular bodies (MVBs) and promotes the formation of MVBs during ferroptosis. Upon fusion of MVBs with the plasma membrane, ferritin-containing exosomes are released into the extracellular space, thus resulting in decreased ferroptosis sensitivity (Brown et al., 2019). PROM2 might interact with the ESCRT-III complex, which promotes membrane repair in RCD, including ferroptosis (Dai et al., 2020). The relationship between PROTO2 and ESCRT-III during ferroptosis needs to be confirmed in the future.

### Solute Carrier Family 40 Member 1

Solute carrier family 40 member 1 (SLC40A1/ferroportin/FPN1) is the only known transmembrane exporter of non-heme iron. Hepcidin antimicrobial peptide (HAMP) is a peptide hormone secreted by the liver that binds to SLC40A1 and induces internalization of SLC40A1 for degradation. It is generally accepted that Fe^2+^ transported out of the cell by SLC40A1 need to be oxidized by ferroxidase (e.g., ceruloplasmin [CP]) to facilitate Fe^3+^ loading on TF. Mutations in SLC40A1 in humans were first reported in 2001 and were found to cause autosomal dominant hemochromatosis (also known as ferroportin disease) (Njajou et al., 2001). Patients with an N144D mutation in the SLC40A1 gene have experienced parenchymal iron loading and cirrhosis. Asparagine 144 may also be important for the function of SLC40A1, because two other disease-causing mutations, N144H (Njajou et al., 2001) and N144T (Arden et al., 2003), have also been identified in autosomal dominant hemochromatosis. The global knockout of SLC40A1 in mice leads to embryonic lethality, while the selective knockout of SLC40A1 in postnatal intestine results in severe iron deficiency (Donovan et al., 2005). Erastin induces the downregulation of SLC40A1 expression in SH-SY5H neuroblastoma cells, which can be reversed by ferroptosis inhibitors, such as ferrostatin-1, an iron chelator, or N-acetyl cysteine (NAC) (Geng et al., 2018). The knockdown of SLC40A1 by RNAi or HAMP-mediated downregulation of SLC40A1 promotes erastin-induced ferroptosis, while ponasterone (a SLC40A1 inducer) impedes this process (Geng et al., 2018). Similarly, the knockdown of SLC40A1 enhances oxygen-glucose deprivation and reoxygenation (OGd/R)-induced ferroptosis in testicular ischemia-reperfusion (I/R) injury sertoli cells (Li L. et al., 2018). In contrast, the overexpression of SLC40A1 or treatment with ponasterone rescues OGd/R-induced ferroptosis by decreasing iron accumulation in sertoli cells (Li L. et al., 2018).

The ferroptosis suppression function of CP is dependent on SLC40A1 (Shang et al., 2020). The depletion of CP promotes erastin- and RSL3-induced ferroptotic cell death, whereas the overexpression of CP suppresses ferroptosis in hepatocellular carcinoma cells (Shang et al., 2020). Intraperitoneal CP treatment has a protective effect on ferroptotic damage after ischemic stroke in mice (Tuo et al., 2017). Collectively, these findings indicate that SLC40A1/CP acts as a negative regulator of ferroptosis.

### Heme Oxygenase 1

Heme, derived primarily from hemoglobin and myoglobin, is a major source of dietary iron in mammals. Heme oxygenase 1 (HMOX1/HO-1) catabolizes heme into three products: carbon monoxide (CO), biliverdin, and free iron. Biliverdin is then reduced to bilirubin by biliverdin reductase (BLVR). HMOX1 can be induced not only by heme but also by a variety of stimuli, such as cytokines, endotoxin, heat shock, and heavy metals, leading to speculation that HMOX1 may play a role in maintaining redox homeostasis. HMOX1 knockout mice have anemia and iron accumulation in liver and kidney, which is associated with oxidative damage and tissue injury. An example of human HMOX1 deficiency was found in a 6-year-old boy suffering from growth retardation, anemia, leukocytosis, thrombocytosis, coagulation abnormalities, and hyperlipidemia (Kawashima et al., 2002), indicating an important role of HMOX1 in human health.

Heme oxygenase 1 plays a dual role in ferroptosis induction. On the one hand, erastin induces the expression of HMOX1, which may promote ferroptosis in HT1080 fibrosarcoma cells. The HMOX1 inhibitor zinc protoporphyrin IX (ZnPP) prevents erastin-induced ferroptotic cell death, whereas the HMOX1 inducer hemin accelerates erastin-induced ferroptosis in HT1080 cells (Kwon et al., 2015). Furthermore, CO-releasing molecules also promote erastin-induced ferroptotic cell death (Kwon et al., 2015), indicating that CO produced by HMOX1 may be an endogenous pro-ferroptosis molecule of ferroptosis. Consistently, the knockout of HMOX1 suppresses, whereas the overexpression of HMOX1 promotes, ferroptotic cancer cell death induced by erastin (Kwon et al., 2015) or BAY 11-7085 (NFKB inhibitor alpha [NFKBIA/IKBA] inhibitor) (Chang et al., 2018). In addition to its affect in cancer models, the inhibition of HMOX1 by ZnPP protects against doxorubicin-induced ferroptotic damage in heart (Fang et al., 2019). On the other hand, HMOX1 also has the ability to inhibit ferroptosis in some cases. For example, erastin and RSL3 treatment increases the expression of HMOX1 in renal proximal tubular cells (Adedoyin et al., 2018). The knockout of HMOX1 increases erastin- or RSL3-induced cell death in renal proximal tubular cells (Adedoyin et al., 2018) or liver cancer cells (Sun et al., 2016b). Thus, the role of HMOX1 in ferroptosis may depend on the context.

### Poly(RC) Binding Proteins

Poly(RC) binding proteins (PCBPs) are iron chaperones that deliver Fe^2+^ to different proteins through a metal-mediated, protein-protein interaction. The targets of PCBP1 and PCBP2 include iron-dependent Egl-9 family hypoxia-inducible factor (EGLN/PHD) and hypoxia-inducible factor 1 subunit alpha inhibitor (HIF1AN/FIH1) that play a role in regulating the transcriptional activity of hypoxia-inducible factor 1 subunit alpha (HIF1A/HIF1α) (Nandal et al., 2011). The depletion of PCBP1 or PCBP2 in cells leads to the loss of EGLN activity, while the addition of excess Fe^2+^ or purified Fe-PCBP1 restores the activity of EGLN (Nandal et al., 2011). As an iron chaperone in the cytosol, PCBP1 directly binds Fe^2+^ and delivers it to ferritin. Consequently, the depletion of PCBP1 suppresses ferritin iron loading and increases cytosolic iron pools in human cells. Interestingly, PCBP2, but not PCBP1, binds to and receives iron from the iron importer SLC11A2 or transfers iron to the iron exporter SLC40A1 (Yanatori et al., 2014). The silencing of PCBP2 expression suppresses SLC11A2-dependent iron uptake and SLC40A1-dependent iron export, indicating that PCBP2 acts as a regulator of iron transport (Yanatori et al., 2014). Moreover, HMOX1 can bind to PCBP2 in the presence of heme, whereas iron-loaded PCBP2 loses its affinity for HMOX1, supporting the role of PCBP2 in heme catabolism and iron transport metabolon. PCBP1 and PCBP2 are essential for mouse embryonic development, but they may play distinct roles in organism function. *Pcbp1*-null embryos are rendered non-viable in the peri-implantation stage (4.5 to 8.5 days postcoitum). Although the differentiation of red blood cells and megakaryocytes is impaired, *Pcbp2*-null embryos undergo normal development until midgestation (12.5 to 13.5 days postcoitum) (Ghanem et al., 2016). Mice heterozygous for either Pcbp1 or Pcbp2 null alleles display a slight reduction in initial postpartum weight (Ghanem et al., 2016).

Poly(RC) binding proteins 1 has been shown to limit ferroptosis in hepatocytes (Protchenko et al., 2020). Mice lacking *Pcbp1* in hepatocytes show defects in iron homeostasis and develop liver disease with hepatic steatosis, inflammation, and degeneration (Protchenko et al., 2020). Transcriptome analysis reveals the activation of lipid biosynthetic and oxidative stress response pathways, including the anti-ferroptotic regulator GPX4. Although PCBP1-deleted hepatocytes are iron-deficient, the supplementation of iron dose doesn’t inhibit the hepatic steatosis, indicating that hepatic steatosis may not require iron deficiency. Paradoxically, hepatocytes lacking PCBP1 show increased redox activity and unchaperoned iron, therefore causing lipid peroxidation (Protchenko et al., 2020). Restricting dietary iron and the supplementing of antioxidant vitamin E can prevent hepatic steatosis associated with ferroptosis (Protchenko et al., 2020). Overall, these findings suggest that PCBP1 plays a complex role in the regulation of ferroptosis-related liver disease.

### Solute Carrier Family 25 Member 37, and Member 28

Solute carrier family 25 member 37 (SLC25A37/mitoferrin-1) and solute carrier family 25 member 28 (SLC25A28/mitoferrin-2) are the key mitochondrial iron importers for heme and iron-sulfur (Fe-S) cluster biogenesis. Due to the difference in turnover between SLC25A37 and SLC25A28, SLC25A37 is highly expressed in erythroid cells, whereas SLC25A28 is ubiquitously expressed in various cells. The loss of *Slc25a37* in mice leads to embryonic lethality, indicating an important role of SLC25A37 in development (Troadec et al., 2011). The selective deletion of SLC25A37 in adult hematopoietic tissues leads to severe anemia, suggesting that the lethality of SLC25A37 depletion might be due to defects in hematopoiesis (Troadec et al., 2011). Interestingly, the selective deletion of SLC25A37 in hepatocytes has no phenotype under normal conditions, but it can cause protoporphyria and hepatotoxicity in delta aminolevulinic acid (a precursor of porphyrin biosynthesis)-fed animals (Troadec et al., 2011). These findings indicate a tissue- and stress-dependent role of SLC25A37 in diseases.

The activity of SLC25A28 in ferroptosis is regulated by the bromodomain-containing protein 7 (BRD7) tumor protein p53 (TP53) pathway. In hepatic stellate cells, ferroptosis inducers increase the expression of BRD7 by inhibiting its proteasome degradation (Zhang et al., 2020). Upregulated BRD7 further promotes mitochondrial translocation of TP53 by directly binding to its N-terminal transactivation domain. Mutations of serine 392, which are necessary for TP53 mitochondrial translocation, block the binding of BRD7 to TP53 and subsequent ferroptosis induction. Importantly, mitochondrial TP53 interacts with SLC25A28 and enhances the activity of SLC25A28, leading to the mitochondrial accumulation of iron and mitochondria ETC-mediated ROS. The knockdown of SLC25A28 impairs BRD7-TP53 signaling-mediated ferroptotic cell death, whereas the overexpression of SLC25A28 facilitates ferroptosis. The PINK1-PARK2 (critical mediators of mitophagy) pathway mediates the degradation of SLC25A37 and SLC25A28, therefore increasing mitochondrial iron accumulation (Li C. et al., 2018). However, the function of mitophagy in ferroptosis remains uncertain. Due to the depletion of mitochondria, parkin-overexpressed HT1080 cells are less sensitive to ferroptosis caused by cystine starvation or erastin (Gao et al., 2019). The pharmacological inhibition of ETC also suppresses ROS production and subsequent lipid peroxidation and ferroptosis (Gao et al., 2019). Taken together, these findings indicate that both the ubiquitin-proteasome system (UPS) and autophagy pathways play a role in regulating mitochondrial iron-dependent ferroptosis by affecting the stability of mitochondrial iron importers.

### CISD1 and CISD2

NEET proteins (also known as CISD proteins) belong to the Fe-S protein family and are characterized by a unique CDGSH motif in their Fe-S cluster-binding domain. There are three members of CISD, namely CDGSH iron sulfur domain 1 (CISD1/mitoNEET), CDGSH iron sulfur domain 2 (CISD2/NAF1), and CDGSH iron sulfur domain 3 (CISD3). CISD1 and CISD3 are outer mitochondrial membrane proteins, whereas CISD2 is mainly located in the endoplasmic reticulum (ER) and mitochondria-associated ER membranes. CISD1 and CISD2 promote tumor growth and metastasis by regulating mitochondrial iron and ROS metabolism. CISD1 and CISD2 were discovered as unexpected targets for the peroxisome proliferator activated receptor gamma (PPARG) agonist pioglitazone (Colca et al., 2004). Pioglitazone is an anti-diabetic drug used in patients with type II diabetes, which also stabilizes CISD1 and CISD2.

As expected, increased CISD1 expression suppresses erastin-induced ferroptosis in human hepatocellular carcinoma cells by limiting mitochondrial iron uptake (Yuan et al., 2016a). Moreover, the knockdown of CISD1 by RNAi increases, whereas stabilization of CISD1 by pioglitazone inhibits, mitochondrial iron uptake and subsequent ferroptosis in response to erastin (Yuan et al., 2016a). Similar to CISD1 as a repressor of ferroptosis, CISD2 is associated with resistance to sulfasalazine-induced ferroptosis in head and neck cancer (Kim et al., 2018). Gene inhibition of CISD2 increases mitochondrial iron accumulation and restores the sensitivity of ferroptosis-resistant cells to sulfasalazine-induced cell death (Kim et al., 2018). In addition to inhibiting ferroptosis, pioglitazone also has the ability to enhance sulfasalazine- or buthionine sulfoximine (BSO)-induced ferroptosis (Kim et al., 2018; Homma et al., 2020). The opposing effects of pioglitazone on ferroptosis in different situations may be due to the difference in cellular localization or function between CISD1 and CISD2 and unknown targets.

### Heat Shock Protein Family B Small Member 1

Heat shock proteins (HSPs) are a class of functionally related stress proteins. When cells are subjected to high temperature or other environmental stress, the expression of HSPs is upregulated to prevent cell damage and death. In addition to helping proteins fold normally, several HSPs also have the ability to regulate iron metabolism. In particular, the expression of heat shock protein family B small member 1 (HSPB1, a member of small HSPs) is increased in erastin-induced ferroptosis (Sun et al., 2015). Protein kinase C (PRKC) further mediates HSPB1 phosphorylation, which stabilizes the actin cytoskeleton (Sun et al., 2015). Finally, a larger actin cytoskeleton inhibits iron uptake and subsequent lipid peroxidation and ferroptosis (Sun et al., 2015). In addition, HSPB1 can inhibit TFRC-mediated iron uptake (Chen et al., 2006), which may also help fight ferroptosis. Collectively, increased HSPB1 expression can promote ferroptosis resistance by blocking iron uptake.

### Aconitase 1 and Iron-Responsive Element Binding Protein 2

**FIGURE 2:**
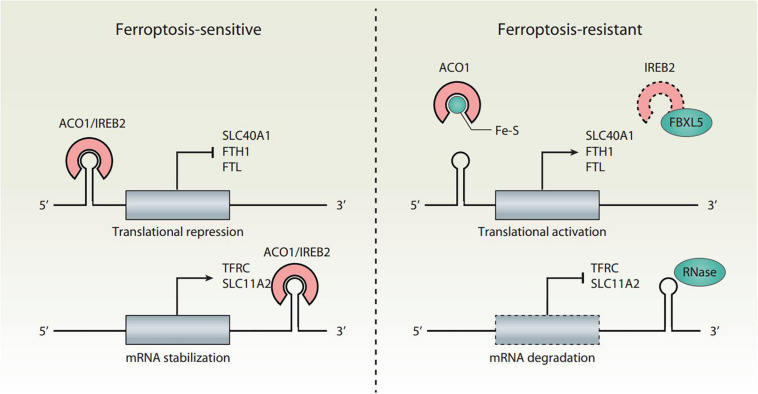
Translational regulation of iron homeostasis in ferroptosis. Ferroptosis is tightly regulated by IRPs (ACO1 and IREB2) at the translational level. The binding of IRPs to IREs leads to increased ferroptosis sensitivity through the translation suppression of SLC40A1, FTH1, and FTL, as well as the translation activation of TFRC and SLC11A2. The conversion of ACO1 into cytosolic aconitase blocks its binding to IREs. IREB2 is negatively regulated by FBXL5-mediated proteasomal degradation. Inhibition of the translation activity of ACO1 or the degradation of IREB2 induces a decrease in iron levels and renders cells resistant to ferroptotic cell death.

Cellular iron homeostasis is tightly regulated at the posttranscriptional level by iron regulatory proteins (IRPs), including aconitase 1 (ACO1/IRP1) and iron-responsive element binding protein 2 (IREB2/IRP2), to adapt to a change of iron levels (Figure 2). ACO1 is a Fe-S cluster protein that exists in two forms. When iron is abundant, ACO1 exists in the Fe-S cluster in the form of cytoplasmic aconitase. When iron is lacking, ACO1 presents in the Fe-S cluster as a regulator of translation. Unlike ACO1, IREB2 is mainly regulated by protein degradation. IREB2 is degraded when iron is excessive, and stable when iron is deficient (Iwai et al., 1998). The iron-responsive elements (IREs) located in the untranslated regions (UTRs) of mRNAs are conserved hairpin structures and can be targeted by IPRs. Depending on the iron charge, the main iron metabolism mRNA is regulated by IRPs, including the genes involved in iron import (e.g., TFRC and SLC11A2), storage (e.g., FTH1 and FTL), and export (e.g., SLC40A1). However, the combination of IRP and IRE located in the 5′ UTR and 3′ UTR has the opposite effect. The binding of IRPs to 5′ IRE leads to the inhibition of translation of mRNA, whereas the binding to 3′ IRE causes the promotion of translation of mRNA by inhibiting the degradation of mRNA. For example, under the condition of iron deficiency, IRPs bind to the 3′ IREs of TFRC and SLC11A2 mRNAs and the 5′ IREs of SLC40A1 and FTH1/FTL mRNAs. As a result, IRP decreases the synthesis of TFRC and SLC11A2, and increases the synthesis of SLC40A1 and FTH1/FTL mRNAs. An excess of cytosolic iron results in translational inhibition of TFRC and SLC11A2, and translational induction of SLC40A1 and FTH1/FTL.

Both ACO1 and IREB2 have an impact on ferroptosis sensitivity (Figure 2). The knockdown of NFS1 cysteine desulfurase (the biosynthetic enzyme of the Fe-S cluster) makes cancer cells susceptible to ferroptosis (Alvarez et al., 2017). Mechanistically, NFS1 suppression activates the iron starvation response in an ACO1-dependent manner, therefore upregulating the expression of TFRC and downregulating the expression of FTH1 (Alvarez et al., 2017). Silencing IREB2 confers protection against erastin-induced ferroptosis, but not against other forms of cell death induced by staurosporine, rotenone, rapamycin, MG132, camptothecin, or thapsigargin (Dixon et al., 2012). In contrast, the knockdown of F-box and leucine-rich repeat protein 5 (FBXL5) or the E3 ubiquitin ligase-mediated proteasomal degradation of IREB2 enhances erastin-induced ferroptosis (Dixon et al., 2012). Therefore, the dysregulation of the IRP system can lead to iron-related effector changes, thereby regulating ferroptosis.

### Transcription Factors

Several transcription factors regulate ferroptosis by controlling the expression of genes involved in iron-related metabolism. Among them, nuclear factor erythroid 2-like 2 (NFE2L2/NRF2) is the key transcription factor that regulates cytoprotective responses to ferroptotic damage. One mechanism of its anti-ferroptotic effect is the upregulation of several genes involved in iron metabolism, such as FTH1 (Sun et al., 2016b), SLC40A1, HMOX1 (Shin et al., 2018; Fang et al., 2019), and metallothionein 1G (MT1G) (Sun et al., 2016a). BTB domain and CNC homolog 1 (BACH1) is a heme-binding transcription factor involved in the regulation of the oxidative stress and heme/iron-related metabolic pathways. The activation of BACH1 enhances erastin-induced ferroptosis through transcriptional downregulation of FTH1, FTL, and SLC40A1 (Nishizawa et al., 2020). The transcription factor metal regulatory transcription factor 1 (MTF1) regulates the expression of several iron-related genes, such as FTH1, FTL, and SLC40A1, and is responsible for ATM serine/threonine kinase inhibition-rescued ferroptosis (Chen P.H. et al., 2020). Cell-to-cell contact can protect cancer cells from ferroptosis by inhibiting the expression of TFRC mediated by transcription factor yes-associated protein 1 (YAP1) (Wu et al., 2019). Heat shock transcription factor 1 (HSF1) is required for erastin-induced expression of HSPB1, which inhibits ferroptosis by blocking cytoskeleton-mediated iron uptake as discussed above (Sun et al., 2015). These findings suggest that multiple stress-related transcription factors play a context-dependent role in ferroptosis.

In addition, hypoxia-inducible factor (HIF) also plays a complex role in ferroptosis, which is regulated by EGLNs (oxygen and iron-dependent enzymes). Under normal oxygen conditions, EGLNs hydroxylate the HIFα subunit, which facilitates its interaction with the von Hippel-Lindau E3 ubiquitin ligase complex and ultimately leads to the proteasome degradation of the HIFα subunit. Under conditions of hypoxia or iron depletion, the activity of EGLNs is inhibited, leading to the stabilization of the HIF subunit, and at the same time the heterodimerization of the α and β subunits of HIF. HIF1 or HIF2 regulates many genes involved in iron homeostasis, such as TFRC, SLC11A2, SLC40A1, CP, HMOX1, and HAMP. Stabilizing HIF1A by hypoxia or cobalt chloride inhibits ferroptosis (Yang et al., 2019), whereas endothelial PAS domain protein 1 (EPAS1/HIF2A) promotes ferroptosis in certain cancer cells (Zou et al., 2019). However, the key downstream genes (not limited to iron metabolism) responsible for HIF-mediated regulation of ferroptosis still need to be further researched.

## Therapeutic Implications of Iron Chelators

Impaired ferroptotic pathways are implicated in various pathologic conditions (e.g., I/R injury and infection) and diseases (e.g., cancer and neurodegenerative diseases) (Xie et al., 2016a; Stockwell et al., 2017). Iron chelators are currently used to treat iron overload diseases. Numerous studies have shown that iron chelators can block ferroptosis *in vitro* and *in vivo*, as described below.

### Deferoxamine

Deferoxamine (DFO) is a natural product isolated from the soil bacterium *Streptomyces pilosus*, and used in the treatment of iron overload patients or aluminum poisoning. As an iron chelator, DFO is a polar molecule with low membrane permeability, which can enter cells through endocytosis. It reacts with Fe^3+^, especially Fe^3+^ in the form of methanesulfonate, and generates a stable octahedral coordination compound, feroxamine, which can be eliminated by the kidneys. DFO has also been shown to stabilize HIF1A by inhibiting the activity of iron-dependent EGLNs, or induce the transcriptional upregulation of HIF1A (Wang and Semenza, 1993). Since DFO was first discovered to have an inhibitory effect on ferroptosis caused by erastin and RSL3, DFO is the drug most widely used to inhibit lipid peroxidation-mediated ferroptosis under various conditions (Dixon et al., 2012). In addition to treating iron overload, DFO also has potential antitumor activity against a variety of cancers *in vivo* and *in vitro* (Buss et al., 2003). However, ferroptosis plays a dual role in cancer biology. Notably, DFO is poorly absorbed and cleared quickly, resulting in ineffective oral administration. Therefore, increasing DFO absorption and metabolic stability is a challenge for its application.

### Deferasirox

Deferasirox is a membrane-permeable iron chelator and the first oral medication approved by the Food and Drug Administration (FDA) for chronic iron overload in the body caused by multiple blood transfusions. It can also be used to treat chronic iron overload in patients with certain blood diseases that do not require blood transfusion (non-transfusion-dependent thalassemia). Deferasirox is a tridentate ligand selective for Fe^3+^ that binds Fe^3+^ at a ratio of 2:1 to form a stable complex. The deferasirox-iron complex is excreted through the kidneys. Unlike iron, deferasirox has a low affinity for zinc and copper. Moreover, deferasirox has a property of inhibiting nuclear factor kappa B (NFKB) (Messa et al., 2010), indicating that deferasirox may be used for limiting inflammation responses in immune cells. Indeed, deferasirox and DFO inhibit hemin-induced ferroptotic cell death and ROS generation in human monocytes (Imoto et al., 2018), thereby increasing the possibility that iron-dependent ferroptosis is involved in activating inflammation.

### Deferiprone

Deferiprone is an oral iron chelator approved by the FDA. When blood transfusion causes iron overload, it can be used as a second-line drug for thalassemia syndrome. Deferiprone combines with Fe^3+^ in a ratio of 3:1 to form a stable complex, which is then eliminated in the urine. Deferiprone is selective for iron, but has a relatively low affinity for other metals (e.g., zinc, copper, and aluminum). Compared to DFO and deferasirox, deferiprone is most effective in the chelation of cardiac iron and equivalent to DFO in the chelation of liver iron. In differentiated Lund human mesencephalic cells, deferiprone is able to specifically inhibit erastin- and glutamate-induced ferroptosis (Do Van et al., 2016). However, whether deferiprone can protect against ferroptosis-mediated tissue damage *in vivo* is unclear.

### Ciclopirox

Ciclopirox is a synthetic broad-spectrum antifungal drug that has been approved by the FDA for the topical dermatological treatment of epidermal mold. This agent is particularly effective in treating tinea versicolor. In addition, ciclopirox has also been shown to exhibit anti-inflammatory effects in human polymorphonuclear cells. The antibacterial and anti-inflammatory properties of ciclopirox may be due to its high affinity for trivalent cations (e.g., Fe^3+^ and Al^3+^), and Fe^3+^ and Al^3+^ are essential cofactors in enzymes. For example, ciclopirox exhibits anti-inflammatory activity by inhibiting the activity of iron-containing arachidonate 5-lipoxygenase (ALOX5) and prostaglandin endoperoxide synthase (PTGS/cyclooxygenase) (Hanel et al., 1991). Ciclopirox is capable of inhibiting ferroptotic cell death, but whether this anti-ferroptosis activity of ciclopirox depends on the inhibition of ALOX5 or PTGS activity is unclear (Dixon et al., 2012).

### Dexrazoxane

Dexrazoxane is a cyclic derivative of EDTA that easily penetrates cell membranes. It is an orphan drug approved by the FDA and can be used in protecting the heart from the cardiotoxic effects of anthracyclines, such as doxorubicin. The precise cardioprotective mechanism of dexrazoxane is not fully understood, but several mechanisms have been proposed. First, the cardioprotective activity of dexrazoxane appears due to the inhibition of the formation of a toxic iron anthracycline complex. Second, dexrazoxane is a pro-drug converted intracellularly to a ring-opened bidentate iron-chelating agent that interferes with iron-mediated ROS production. Third, the cardioprotective activity of dexrazoxane also involves the inhibition of topoisomerase II-mediated DNA double-strand breaks. Fourth, dexrazoxane or ferrostatin-1 inhibits ferroptosis in a doxorubicin-induced cardiac injury model (Fang et al., 2019). Of note, other studies suggest that Mito-FerroGreen (MFG, a fluorescence indicator specifically chelating Fe^2+^ in mitochondria), but not dexrazoxane and DFO, prevents doxorubicin-induced lipid peroxidation and mitochondria-dependent ferroptosis in cardiomyopathy (Tadokoro et al., 2020), indicating that mitochondrial iron may play a major role in mediating ferroptotic heart damage.

### Baicalein

Baicalein is a flavonoid extracted from *Scutellaria baicalensis Georgi* and has free 5,6,7- hydroxyl groups that form complexes with iron in a stoichiometry of 1:1. Compared to a natural product library of 143 compounds, baicalein showed the strongest protection against erastin-induced ferroptotic cell death in human pancreatic cancer cell lines (Xie et al., 2016b). Baicalein reverses erastin-induced intracellular iron accumulation, GSH depletion, and GPX4 degradation (Xie et al., 2016b). In addition to cancer cells, baicalein exerts neuroprotective effects on posttraumatic epileptic seizures by repressing ferroptotic cell death in mouse models (Li et al., 2019). Baicalein is also effective in inhibiting ferric ammonium citrate (FAC)-induced HT22 hippocampal neuron damage (Li et al., 2019). Moreover, baicalein markedly decreases iron-induced lipid peroxidation and inhibits the expression of arachidonate 12-lipoxygenase (ALOX12) or arachidonate 15-lipoxygenase (ALOX15) in HT22 cells (Li et al., 2019). In addition to its iron chelator activity, baicalein also inhibits the activity of several oxidases (e.g., ALOXs), which may also contribute to its anti-ferroptosis effect (Yang et al., 2016).

### Other Iron Chelators

Another Fe^2+^ chelator is 2,2′-bipyridine, which is membrane-permeant and known to sequester iron from labile iron pool (LIP) in cells. The 2,2′-bipyridine may enter mitochondria and chelate mitochondrial iron, therefore decreasing the generation of ROS (Chang et al., 2016). As expected, 2,2′-bipyridine is an inhibitor of ferroptosis by decreasing iron-dependent lipid peroxidation (Dixon et al., 2012). Additionally, 1,10-phenanthroline is also an Fe^2+^ chelating agent, with coordination properties similar to 2,2′-bipyridine. The 1,10-phenanthroline blocks zero-valent iron nanoparticle-induced mitochondrial ROS accumulation and subsequent ferroptosis *in vitro* (Huang et al., 2019).

## Conclusion and Perspectives

Excess of free reactive iron can cause various types of cell death, including a recently recognized type, ferroptosis. Although the core molecular effector of ferroptosis is unclear, ferroptosis is induced by the activation of iron-dependent lipid peroxidation (Kuang et al., 2020). Significant progress has been made in dissecting the mechanisms that lead to lipid peroxidation and how antioxidant systems or stress proteins regulate ferroptosis. However, the exact contribution of iron-mediated ROS production and iron-containing enzymes in ferroptosis and its dynamic relationship with other types of RCDs is still poorly understood. In ferroptosis, whether any proteins act downstream of lipid peroxidation and how they are affected by iron homeostasis remains an enigma. Further studies are needed to determine and develop optimal targeted therapy for the specific ferroptosis-associated disease. In addition, further evidence is needed to show improved prognosis when ferroptotic cell death is selectively blocked. Nevertheless, recent work has begun to clarify the involvement of ferroptosis in different aspects of diseases (e.g., cancer, neurodegenerative diseases, and I/R injury-related diseases). Generally, it can be expected that treatment strategies aimed at suppressing ferroptotic signals and pathways will benefit iron overload diseases. In particular, the use of iron chelating agents as potential therapeutic agents has been widely considered in the treatment of ferroptotic diseases, although these iron chelators have different pharmacokinetic and metabolic properties. Despite the great potential value, further *in vivo* studies must be conducted to clarify the molecular mechanism and mode of action of these iron chelators. There are still challenges to develop a candidate iron chelator with relatively low toxicity and high efficiency. Overall, it is necessary to further study the mechanism of iron-dependent lipid peroxidation in order to establish more accurate targets for ferroptosis-associated diseases (Chen X. et al., 2020). Whether iron overload is enough to cause ferroptosis in various cells or tissues also needs to be investigated.

## Author Contributions

XC, CY, RK, and DT conceptualized and wrote the manuscript. All authors contributed to the article and approved the submitted version.

## Conflict of Interest

The authors declare that the research was conducted in the absence of any commercial or financial relationships that could be construed as a potential conflict of interest.
